# The Burden of Blood-Pressure-Related Cardiovascular Mortality in Mexico

**DOI:** 10.1155/2014/427684

**Published:** 2014-02-10

**Authors:** Dora E. Cortés-Hernández, Krista J. Lundelin, Esteban Picazzo-Palencia, Juan J. de la Cruz, José J. Sánchez, José R. Banegas

**Affiliations:** ^1^Department of Public Health and Preventive Medicine, School of Medicine and Center for Research and Development on Health Science, Universidad Autónoma de Nuevo Leon, 64460 Monterrey, NL, Mexico; ^2^Department of Preventive Medicine and Public Health, Universidad Autónoma de Madrid/IdiPAZ, CIBER in Epidemiology and Public Health (CIBERESP), 28029 Madrid, Spain; ^3^Institute for Social Research, Center for Research and Development on Health Science, Faculty of Accounting and Business Administration, Universidad Autónoma de Nuevo León, 64460 Monterrey, NL, Mexico

## Abstract

This study shows that in Mexico, a country at an advanced stage in the epidemiologic transition, with the national burden of disease dominated by noncommunicable diseases, elevated blood pressure is a major clinical and public health problem. 31.7% of the Mexican individuals aged 50 and over had systolic hypertension, and 47.3% were at systolic prehypertensive levels. Also, approximately half of all cardiovascular deaths that occurred annually in the population of Mexico aged ≥50 years are attributable to above optimal levels of systolic blood pressure. We think these estimates may help order health priorities in Mexico (and other middle-income countries) at a time when the costs of medical care take a considerable share of the gross national product in most countries.

## 1. Introduction

Among middle-age and old-age adults, blood pressure (BP) is strongly and directly associated with mortality in the absence of a clear threshold until at least 115/75 mmHg [1]. Not surprisingly, high BP is the most important cause of stroke and one of the most important risk factors for coronary heart disease (CHD) [1–3]. In fact, randomized controlled trials have confirmed that treatment of hypertension reduces the risk of cardiovascular disease (CVD) events at all ages [4, 5]. Additionally, a recent meta-analysis has shown that the risk of stroke is significantly reduced with antihypertensive therapy in cohorts with prehypertensive baseline BP levels [6] and, further, that lifestyle modifications also play a significant role in reducing the risk of cardiovascular events among prehypertensive patients [7].

High BP and CVD are major causes of death and disability worldwide [8–11]. Although safe and effective strategies for the prevention and control of high BP have been widely available in many countries for over 50 years, overall BP control rates remain suboptimal in most countries [12–17]. Mexico is a country at an advanced stage in the epidemiologic transition with the burden of disease at the national level dominated by noncommunicable diseases and related risk factors, including very high rates of heart disease, stroke, and hypertension [18]. In fact, CHD and stroke are the second and third single leading causes of death in Mexico [19]. Surprisingly, these excessive rates of noncommunicable diseases, mostly CVD-related, exist in the context of significant progress regarding the universal health coverage system [20]. Although recent work has reported data on the prevalence and control of hypertension in Mexico [21, 22], no information on the current burden of cardiovascular mortality attributable to relevant past levels of hypertension and prehypertension has been reported. This information would inform public health authorities about the real impact of elevated BP on the main causes of death at the population level.

Thus, this study estimates the current burden of CVD mortality attributable to past levels of higher-than-optimal systolic BP (SBP) in the Mexican population, specifically among people 50 years and over, where SBP is the main predictor of CVD risk and most CVD deaths are expected to occur [1, 2]. In particular, we have taken into account a reasonable time lag of at least a few years from exposure to ensuing mortality burden [1, 23, 24]. In addition to informing public policy in this country, this evidence could provide valuable information to other low- and middle-income countries also experimenting epidemiological transition, where burden of BP-related disease does not seem to be a public health priority yet.

## 2. Methods

### 2.1. Survey Design

Our study is based on the 2005-2006 Mexican National Health and Nutrition Survey, a cross-sectional study that collected data from a sample representative of the Mexican population and that was conducted by the Mexican National Institute of Public Health between October 2005 and May 2006. Detailed methods of the study are described elsewhere [25]. In brief, a probabilistic multistage stratified cluster sampling method was used to select 48,600 households in all 32 states of Mexico, of which 47,152 agreed to participate. The sampling process comprised the following steps: (1) random selection of “Areas Geoestadísticas Básicas” (Basic Geostatistical Areas) or AGEB in each of the 32 states with probability proportional to the number of households; (2) selection of AGEB by county with probability proportional to the county's population; (3) selection of six blocks per AGEB with equal probability; (4) selection of six households per block with equal probability; and (5) selection of one individual from each age group (a child, an adolescent, and an adult aged ≥20 years) chosen with equal probability. The survey has power to make distinctions between urban (≥2,500 inhabitants) and rural (<2,500 inhabitants) areas. Sample weights for each participant were calculated in order to adjust for the complex survey design based on the corresponding age and gender distributions as well as on national census information. All study participants gave written informed consent. The study was approved by the Research, Ethics, and Biosecurity committees of the Mexican National Institute of Public Health.

### 2.2. Measurements

Data collection consisted of two home visits by trained health personnel. During the first visit the health interview and anthropometric measurements were obtained, whereas during the second visit blood samples and BP measurement were collected. A structured questionnaire was administered to obtain sociodemographic and personal health data including self-reports of previously diagnosed type 2 diabetes, hypercholesterolemia, tobacco use, alcohol consumption, physical activity, and history of cardiovascular disease.

Anthropometric measures included weight, height, and waist circumference, under standardized conditions. Height was measured to the nearest 0.1 cm using a stadiometer with an error of 5 mm, whereas body weight was measured using a digital scale with an error of 0.1 kg. Body mass index (BMI) was calculated as weight (kg)/height (m^2^); obesity was defined as a BMI ≥ 30 kg/m^2^ [26] and abdominal obesity as waist circumference >90 cm in men and >80 cm in women.

Blood pressure was measured twice, using the dominant arm of seated participants after resting for a minimum of 5 minutes. Calibrated standard mercury sphygmomanometers were used and standardized methods and conditions were followed [27]. The mean of the two BP readings was used for analyses. For this study's purpose, we defined systolic hypertension as SBP ≥ 140 mmHg, regardless of DBP values or treatment status, and higher-than-optimal SBP as SBP ≥ 120 mmHg, and SBP categories followed classification from international and national guidelines [28–31].

### 2.3. Statistical Analysis

For this study, we focused on the 10,004 participants aged 50 years and over with complete data of the total Mexican adults surveyed. Descriptive data are presented as frequencies and percentages for qualitative variables and as mean ± standard deviation for quantitative variables. Differences in percentage of people between hypertension and no hypertension groups were assessed using the *χ* ^2^ test (or Fisher exact test where appropriate). Analyses were performed with the “Survey Data” procedures in STATA v.11.1 (StataCorp. LP, College Station, 2010) to account for the complex sampling design.

Population attributable fraction (PAF) of cardiovascular mortality attributable to higher-than-optimal SBP was calculated for each CVD considered (those for which evidence on a causal relationship with high BP is available), each age group (by decades) and SBP (*i*) category, according to the classical formula [32, 33]: PAF_*i*_ = *P*_*i*_(RR_*i*_ − 1)/(1 + ∑*P*_*i*_[RR_*i*_ − 1]). For the total of SBP categories we used the formula: PAF = ∑_*i*_ *P*_*i*_(RR_*i*_ − 1)/(1 + ∑*P*_*i*_[RR_*i*_ − 1]), where RR_*i*_ stands for relative risk of death for CVD in each age group and SBP level (with respect to the reference category <120 mmHg) and *P* stands for the prevalence of SBP in the study population in each category “*i*” and age group. RR data were drawn from the Prospective Studies Collaboration, a meta-analysis of 61 studies on BP and mortality, including data on 1 million subjects with no prior CVD recorded at baseline [1]. To approximate to the BP categories used in international guidelines and local guidelines in Mexico [28–31], we used the RRs at midpoint for each BP category reported in the Prospective Studies Collaboration. Percentages of SBP categories were drawn from the nationwide survey of Mexico [25] for subjects ≥50 years without previous clinical CVD.

Finally, the number of cardiovascular deaths attributable to each SBP category and within each age group were calculated by multiplying the corresponding PAF by the number of cardiovascular deaths registered in each age group in the Mexican population ≥50 according to official statistics [19]. The following mortality causes were considered CVD deaths: CHD (codes I20–I25) (65,371 deaths), stroke (I60–69, G45) (29,520 deaths), and other cardiovascular diseases (25,677 deaths) including heart failure (I50), hypertensive disease (I10–I15), atherosclerosis (I70), sudden death (I46.1), rheumatic heart disease (I05–I09), and pulmonary embolism (I26) according to the International Classification of Diseases system 10th revision [34].

## 3. Results

### 3.1. Sample Characteristics

41.5% of subjects (4150) were aged 50–59 years old; 30%, 60–69; 20%, 70–79; and 8.5%, 80–89. Fifty-seven percent were women, 28.6% were smokers, 32.6% had obesity (57.3%, abdominal obesity), 15.1% had diabetes, 28.4% were sedentary, and 8.8% reported having a previous CVD event (Table 1). Mean SBP in the study subjects was 132.3 ± 19.3 mmHg, and its stratification by sex, age, and clinical characteristics is shown in Table 1.

### 3.2. Distribution of Systolic Blood Pressure

Overall, 31.7% of individuals had systolic hypertension (SBP ≥ 140 mmHg), for either being untreated or uncontrolled. Some 47.3% of individuals were at systolic prehypertensive levels, because they were either hypertensive with their SBP values controlled or treatment naïve prehypertensive.

The prevalence of systolic hypertension was significantly higher in women, older age, and those with obesity, diabetes or sedentariness than in their counterparts (Table 1), but it was lower among those who were smokers. The percentage of people within the 120–129 mmHg range was generally somewhat higher than that within 130–139 mmHg, except for those with obesity (BMI ≥ 30 kg/m^2^), sedentariness, or diabetes where the opposite occurred (Table 1). Interestingly, prehypertensive categories were generally more frequent than any of the hypertension categories, except for obesity and diabetes again where grade 1 hypertension took over the first place.

### 3.3. Percentage of Cardiovascular Mortality Attributable to High Systolic Blood Pressure

As shown in Table 2, the prevalence of systolic hypertension increased, and the percentage of systolic prehypertensive levels decreased with age until age 80 and then tended to level off. RRs for cardiovascular death rose steeply with increasing levels of SBP, decreased with age, and they were noticeably higher for stroke among individuals under the age of 80 (Table 2).

39–58% of all CHD deaths, 39–75% of all stroke deaths, and 35–59% of other CVD deaths occurred in the population of Mexico aged 50 and over were calculated to be attributable to higher-than-optimal levels of SBP (Table 2). PAFs generally decreased with age and were higher for stroke under age 80. Hypertension categories accounted for the highest part of PAF for all age groups and CVD types.

**Figure 1:**
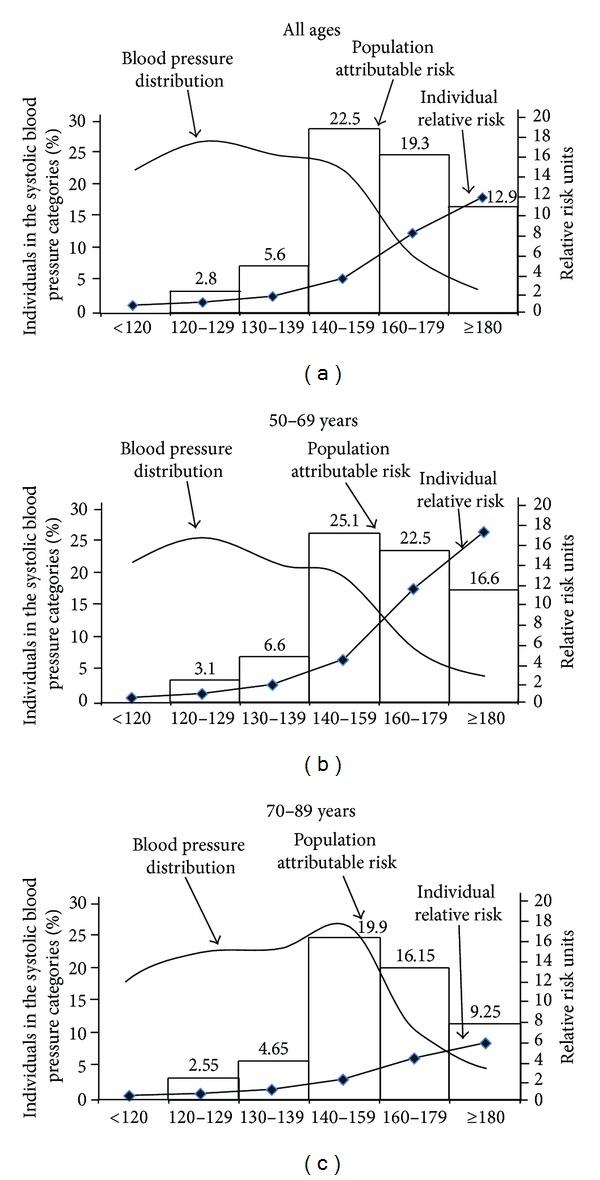
Percent mortality from all cardiovascular diseases attributable to higher-than-optimal systolic blood pressure in the population of Mexico aged 50 and over in 2010. The curve represents the smoothed distribution of systolic blood pressure (SBP) in the 2005-2006 Mexican sample based on SBP categories. The risk line represents the internationally-based relative risk values of cardiovascular mortality corresponding to each SBP category. The numbers at the heads of the bars indicate the percentages of all cardiovascular deaths occurred in Mexico in 2010 attributable to each category of SBP, by age.

The figure shows that whereas RRs increased with progressively higher levels of SBP, population attributable fractions (or risks) increased up to grade 1 systolic hypertension levels and then decreased (Figure 1).

Overall, 63.6% of all CVD deaths occurred in the population of Mexico ≥50 years were attributable to above optimal SBP, 73.9% of all CVD deaths in individuals 50–69 years and 52.6% in those 70–89 years (Figure 1). A much lower but not negligible 7–10% of all these cardiovascular disease deaths that occurred arise from individuals at SBP 120–139 mmHg.

### 3.4. The Absolute Burden of High Systolic Blood Pressure on Cardiovascular Mortality in the Population

A total of 57,896 cardiovascular deaths were attributable to high SBP. This stands for almost half (48%) of all annual cardiovascular deaths that occurred in the Mexican population ≥50 years (47% and 54.7% of all CHD and stroke deaths, resp.) (Table 3). A total of 30,706 of these related deaths were coronary heart disease deaths, 16,161 stroke deaths, and 11,029 deaths from other CVDs. The highest number of related deaths came from hypertension grade 1 and 2 (48,339 or 83.4%), and fell on patients over 70 years (38,598 or 66.6%) (Table 3). Nevertheless, systolic prehypertension (120–139 mmHg) accounted for 16.5% of all related deaths (9,557 deaths), and 33.3% of all related deaths came from individuals aged 50–69 years (19,298 deaths) (Table 3).

## 4. Discussion

This is the first study carried out in Mexico, based on population data representative of the whole population of Mexico, that presents information on the burden of cardiovascular mortality related to elevated SBP in noninstitutionalized people older than 49 years. This study shows that elevated systolic blood pressure is a major public health problem in Mexico. As many as 58,000 cardiovascular deaths in the Mexican population aged 50 and over are attributable to elevated SBP levels, about half of all CVD deaths occurred in those people at present; from these, 8 in ten are due to systolic hypertension and 2 due to systolic prehypertension.

As in a previous study performed in Spain [35], CHD accounted for more BP-related cardiovascular mortality than stroke. However, in both Mexico and Spain, stroke accounted for a higher PAF and percentage of all related deaths. This may be partly due to the relatively higher prevalence of hypertension and prehypertension and poorer BP control in the Mexican population than other more developed countries [21, 36–38], as these characteristics are more strongly related to stroke [39, 40].

One in 3 related deaths were premature deaths (occurring before age of 70 years; mean life expectancy in Mexico was about 75 [41]), amounting to almost 20,000 annual cardiovascular deaths. Estimates of the contribution of raised blood pressure to premature mortality are essential for anticipating how much benefit from prevention could be potentially achieved, especially in economically developing countries, where healthcare resources are scarce and BP-related burden is considerable [9, 11, 42].

Most related deaths came from hypertension, however about 15–20% of related mortality arises from the relatively minority who fall within prehypertensive levels, even in individuals under the age of 70 (data not shown). This is partially consistent with theory from Rose [43] that the graded increase of individual RR of CVD mortality throughout the SBP range is not accompanied by a corresponding increase in the population burden of mortality, since the percentage of individuals decreases steeply above grade 1 systolic hypertension (see Figure 1).

### 4.1. Clinical and Public Health Implications

These findings may have important implications since high SBP is a major clinical and public health problem in Mexico. The huge burden of hypertension-related cardiovascular mortality hints at a worrying combination of high prevalence and poor management of hypertension. In fact, one-third of adult Mexicans are systolic-based hypertensive patients, who are either untreated or uncontrolled despite treatment. Thus, it is reasonable to suggest that a substantial portion of deaths attributable to hypertension are likely to be due to uncontrolled hypertension. Achieving higher levels of BP control in this population would potentially reap significant benefits in the form of important reductions in cardiovascular mortality, thus demonstrating the large harmful impact that hypertension has on the health of an entire population.

However, the figures of the clinical epidemiology of hypertension management (awareness, treatment, and control) are persistently poor in many countries, especially in less developed countries [36, 37]. This suggests that if a substantial impact is to be achieved in the national figures of cardiovascular burden in the population, better detection and treatment of high blood pressure (rather than only hypertension) based on both lifestyle intervention and effective drug prescription should be implemented [30, 44, 45]. In other terms, no matter how big the problem of hypertension is, the burden of cardiovascular mortality arises from the whole range of BP values. Prevention, to be effective, must address the whole range of the problem.

Finally, results from this study may inform related public health policy in other middle-income countries also experimenting epidemiological transition.

### 4.2. Limitations and Strengths

Our results should be interpreted in the context of the study's limitations. First, we may have overestimated the prevalence of hypertension since BP measures used for analyses were based on the average of only two BP readings and given the known fact that BP tends to fall spontaneously over time. Second, the input RRs used to calculate the BP-related CVD mortality, mainly coming from European and American studies, may probably be slightly lower than those in Mexicans, especially those referred to CHD [46]. This may have resulted in some underestimation of related mortality. However, RRs for most risk factors effects seem to be similar across populations [40, 47], indicating that combining and applying international studies to Mexico is reasonably appropriate awaiting for a more accurate estimation from local longitudinal studies. Third, since no RRs for combined values of SBP and DBP were available from the Prospective Studies Collaboration [1], we only used SBP. In our study, exclusion of elevated DBP may have resulted in underestimation of related mortality. Fourth, for calculating PAF we used classical formulae [32, 33], whose validity is maximized in the absence of confounding. To reduce confounding, we controlled the effect of age, the main confounder in the relationship between SBP and CVD mortality, by using age-stratified RRs and BP prevalence. Fifth, we calculated the current burden of CVD mortality related to past exposure to elevated levels of SBP (related burden), instead of the future burden due to current exposure (avoidable burden). Nevertheless, related burden may be interesting, because is likely to be a good predictor of avoidable mortality [23]. To this purpose, we assumed a time lag of about 5 years between exposure to high BP and current mortality, which is obviously subjected to some uncertainty since no clear lag periods have been established. Finally, we have used mortality and no disability as an estimation of burden of disease. Future studies could examine disability-adjusted life years.

It should also be noted that mortality burden attributable to high SBP should be interpreted in the context of other CVD risk factors interacting with BP to produce this burden of deaths; and that even if prevention were successful to avoid disease, death will eventually be only postponed, this nevertheless being worth achieving (healthier and longer life).

Major strengths of this study include the size and characteristics of the database analysed. The National Health and Nutrition Survey of Mexico is larger than similar surveys in most developed countries; it closely represents the Mexican adult population, and it collects objective BP measures. However women were slightly overrepresented in the Mexican National Survey (58.7% versus 52.3% in the general population), due in part to the reduced presence of men in the household during working hours, the age, and sex distribution of the sample which closely resembled the general population (data not shown). Future research will continue to benefit from the data provided from large ongoing national surveys of the Mexican population which enable the surveillance of the clinical epidemiology of hypertension and prehypertension and the impact of BP-related cardiovascular mortality.

## Figures and Tables

**Table 1:** Demographic and clinical characteristics of the sample of Mexican individuals aged 50 and over, according to systolic blood pressure categories.

			SBP category, mmHg						SBP dichotomized, mmHg		
	*N*	SBP	<120	120–129	130–139	140–159	160–179	≥180	<140	≥140	*P *
		Mean ± SD	*n* (%)	*n* (%)	*n* (%)	*n* (%)	*n* (%)	*n* (%)	*n* (%)	*n* (%)	
Total	**10004**	132.3 ± 19.3	**2098 (21.0)**	**2501 (25.0)**	**2234 (22.3)**	**2132 (21.3)**	**770 (7.7)**	**269 (2.7)**	**6833 (68.3)**	**3171 (31.7)**	
Gender											0.008
Male	4299	131.8 ± 18.7	891 (20.7)	1128 (26.2)	978 (22.7)	898 (20.9)	307 (7.1)	97 (2.3)	2997 (69.7)	1302 (30.3)	
Female	5705	132.7 ± 19.7	1207 (21.2)	1373 (24.1)	1256 (22.0)	1234 (21.6)	463 (8.1)	172 (3.0)	3836 (67.2)	1869 (32.8)	
Age, years											<0.001
50–59	4150	128.8 ± 17.6	1077 (26.0)	1150 (27.7)	916 (22.1)	707 (17.0)	231 (5.6)	69 (1.7)	3143 (75.7)	1007 (24.3)	
60–69	3005	133.5 ± 19.1	543 (18.1)	752 (25.0)	685 (22.8)	693 (23.1)	251 (8.4)	81 (2.7)	1980 (65.9)	1025 (34.1)	
70–79	1997	136.8 ± 21.0	314 (15.7)	406 (20.3)	456 (22.8)	525 (26.3)	205 (10.3)	91 (4.6)	1176 (58.9)	821 (41.1)	
80–89	852	134.5 ± 20.2	164 (19.2)	193 (22.7)	177 (20.8)	207 (24.3)	83 (9.7)	28 (3.3)	534 (62.7)	318 (37.3)	
BMI, kg/m^2^											<0.001
<25	2798	128.9 ± 18.9	765 (27.3)	742 (26.5)	577 (20.6)	482 (17.2)	179 (6.4)	53 (1.9)	2084 (74.5)	714 (25.5)	
25–29.9	3944	132.1 ± 19.1	837 (21.2)	998 (25.3)	871 (22.1)	856 (21.7)	284 (7.2)	98 (2.5)	2706 (68.6)	1238 (31.4)	
≥30	3262	135.5 ± 19.3	496 (15.2)	761 (23.3)	786 (24.1)	794 (24.3)	307 (9.4)	118 (3.6)	2043 (62.6)	1219 (37.4)	
Central obesity											<0.0001
No	4276	129.3 ± 18.5	1079 (25.2)	1170 (27.4)	941 (22.0)	747 (17.5)	270 (6.3)	69 (1.6)	3190 (74.6)	1086 (25.4)	
Yes	5728	134.5 ± 19.5	1019 (17.8)	1331 (23.2)	1293 (22.6)	1385 (24.2)	500 (8.7)	200 (3.5)	3643 (63.6)	2085 (36.4)	
Alcohol											0.065
No	5769	132.7 ± 19.7	1202 (20.8)	1420 (24.6)	1276 (22.1)	1215 (21.1)	484 (8.4)	172 (3.0)	3898 (67.6)	1871 (32.4)	
Yes	4235	131.7 ± 18.6	896 (21.2)	1081 (25.5)	958 (22.6)	917 (21.7)	286 (6.8)	97 (2.3)	2935 (69.3)	1300 (30.7)	
Smoking											0.017
No	9125	132.7 ± 19.3	1461 (20.5)	1772 (24.8)	1593 (22.3)	1546 (21.7)	563 (7.9)	204 (2.9)	4826 (67.6)	2313 (32.4)	
Yes	2865	131.4 ± 19.1	637 (22.2)	729 (25.4)	641 (22.4)	586 (20.5)	207 (7.2)	65 (2.3)	2007 (70.1)	858 (29.9)	
Sedentariness											<0.001
No	1924	130.5 ± 18.7	453 (23.5)	496 (25.8)	442 (23.0)	367 (19.1)	131 (6.8)	35 (1.8)	1391 (72.3)	533 (27.7)	
Yes	764	134.6 ± 20.1	142 (18.6)	166 (21.7)	178 (23.3)	176 (23.0)	79 (10.3)	23 (3.0)	486 (63.6)	278 (36.4)	
Diabetes											<0.001
No	8491	131.8 ± 19.2	1840 (21.7)	2177 (25.6)	1876 (22.1)	1754 (20.7)	626 (7.4)	218 (2.6)	5893 (69.4)	2598 (30.6)	
Yes	1513	135.0 ± 19.4	258 (17.1)	324 (21.4)	358 (23.7)	378 (25.0)	144 (9.5)	51 (3.4)	940 (62.1)	573 (37.9)	

SBP: systolic blood pressure. BMI: body mass index. Central obesity: waist circumference >102 com in men and >88 cm in women.

**Table 2:** Cardiovascular relative risk and percent risk attributable to above-optimal systolic blood pressure in the population of Mexico over age 50 in 2010.

Age (years)	SBP (mmHg)	Blood pressure	Coronary heart disease		Stroke		Other CVD	
	Category	%	Relative risk	PAF	Relative risk	PAF	Relative risk	PAF
50–59	<120 (ref)	26.0	1.0	0.0	1.0	0.0	1.0	0.0
	120–129	27.7	1.3	3.6	1.5	3.6	1.3	3.7
	130–139	22.1	1.8	5.8	2.4	6.0	1.8	5.9
	140–159	17.0	3.2	18.1	5.3	21.0	3.2	17.4
	160–179	5.6	6.5	14.7	14.0	20.6	6.5	14.9
	≥180	1.7	8.8	15.6	21.0	23.8	8.8	15.8
	Total	—	—	**57.9**	—	**75.0**	—	**57.5**

60–69	<120 (ref)	18.1	1.0	0.0	1.0	0.0	1.0	0.0
	120–129	25.0	1.3	3.1	1.4	2.6	1.3	3.0
	130–139	22.8	1.7	6.0	2.1	6.2	1.7	5.8
	140–159	23.1	2.8	19.3	4.2	22.3	2.9	19.8
	160–179	8.4	5.2	14.9	9.9	20.5	5.5	15.4
	≥180	2.7	6.9	14.4	14.2	20.9	7.4	15.1
	Total	—	—	**57.7**	—	**72.5**	—	**59.1**

70–79	<120 (ref)	15.7	1.0	0.0	1.0	0.0	1.0	0.0
	120–129	20.3	1.2	2.3	1.3	2.5	1.2	2.5
	130–139	22.8	1.6	6.1	1.8	6.0	1.5	5.6
	140–159	26.3	2.3	17.9	3.2	22.1	2.1	16.6
	160–179	10.3	3.9	13.2	6.5	18.2	3.3	11,5
	≥180	4.6	5.0	10.3	8.8	14.7	4.1	8.8
	Total	—	—	**49.9**	—	**63.5**	—	**45.0**

80–89	<120 (ref)	19.2	1.0	0.0	1.0	0.0	1.0	0.0
	120–129	22.7	1.2	3.3	1.2	3.3	1.1	1.8
	130–139	20.8	1.4	4.0	1.4	4.0	1.4	4.2
	140–159	24.3	1.9	13.7	1.9	13.7	1.8	13.0
	160–179	9.7	2.9	12.5	2.9	12.5	2.6	11.2
	≥180	3.3	3.5	5.5	3.5	5.5	3.1	4.9
	Total	—	—	**39.0**	—	**39.0**	—	**35.0**

SBP: systolic blood pressure. Other CVD: other cardiovascular disease (heart failure, hypertensive disease, atherosclerosis, sudden death, rheumatic heart disease, and pulmonary embolism). PAF: population attributable fraction. Ref: reference category.

**Table 3:** Number and distribution of cardiovascular deaths attributable to above optimal systolic blood pressure (SBP), by age and SBP category in the population of Mexico aged 50 and over in 2010.

	Coronary heart disease *n* (%)	Stroke *n* (%)	Other CVD *n* (%)	All CVD *n* (%)
Age (years)				
50–59	3909 (12.7)	2091 (12.9)	1284 (11.6)	7284 (12.6)
60–69	6483 (21.1)	3370 (20.8)	2161 (19.6)	12014 (20.7)
70–79	8470 (27.6)	5430 (33.6)	2917 (26.4)	16817 (29.0)
80–89	11844 (38.6)	5270 (32.6)	4667 (42.3)	21781 (37.6)
SBP (mmHg)				
120–129	1993 (6.5)	890 (5.5)	589 (5.3)	3472 (6.0)
130–139	3317 (10.8)	1500 (9.3)	1268 (11.5)	6085 (10.5)
140–159	10620 (34.6)	5374 (33.2)	3935 (35.7)	19929 (34.4)
160–179	8694 (28.3)	4771 (29.5)	3121 (28.3)	16586 (28.6)
≥180	6082 (19.8)	3626 (22.4)	2116 (19.2)	11824 (20.4)

Total				
Number	30706 (100)	16161 (100)	11029 (100)	57896 (100)
% of total CVD	47.0	54.7	42.9	48.0

Total: sum of related deaths over all age groups or all SBP categories, for each cardiovascular disease (CVD) type and for all CVD together. % of total CVD: percentage of all cardiovascular deaths registered in the population of Mexico over 50 that were attributable to higher-than-optimal blood pressure (SBP ≥ 120 mmHg). Other cardiovascular disease: heart failure, hypertensive disease, atherosclerosis, sudden death, rheumatic heart disease, and pulmonary embolism.
